# Localization of the Cochlear Amplifier in Living Sensitive Ears

**DOI:** 10.1371/journal.pone.0020149

**Published:** 2011-05-23

**Authors:** Tianying Ren, Wenxuan He, Edward Porsov

**Affiliations:** 1 Oregon Hearing Research Center, Department of Otolaryngology and Head & Neck Surgery, Oregon Health & Science University, Portland, Oregon, United States of America; 2 Department of Physiology, Xi'an Jiaotong University School of Medicine, Xi'an, Shaanxi, People's Republic of China; Tokyo Medical and Dental University, Japan

## Abstract

**Background:**

To detect soft sounds, the mammalian cochlea increases its sensitivity by amplifying incoming sounds up to one thousand times. Although the cochlear amplifier is thought to be a local cellular process at an area basal to the response peak on the spiral basilar membrane, its location has not been demonstrated experimentally.

**Methodology and Principal Findings:**

Using a sensitive laser interferometer to measure sub-nanometer vibrations at two locations along the basilar membrane in sensitive gerbil cochleae, here we show that the cochlea can boost soft sound-induced vibrations as much as 50 dB/mm at an area proximal to the response peak on the basilar membrane. The observed amplification works maximally at low sound levels and at frequencies immediately below the peak-response frequency of the measured apical location. The amplification decreases more than 65 dB/mm as sound levels increases.

**Conclusions and Significance:**

We conclude that the cochlea amplifier resides at a small longitudinal region basal to the response peak in the sensitive cochlea. These data provides critical information for advancing our knowledge on cochlear mechanisms responsible for the remarkable hearing sensitivity, frequency selectivity and dynamic range.

## Introduction

**Figure 1:**
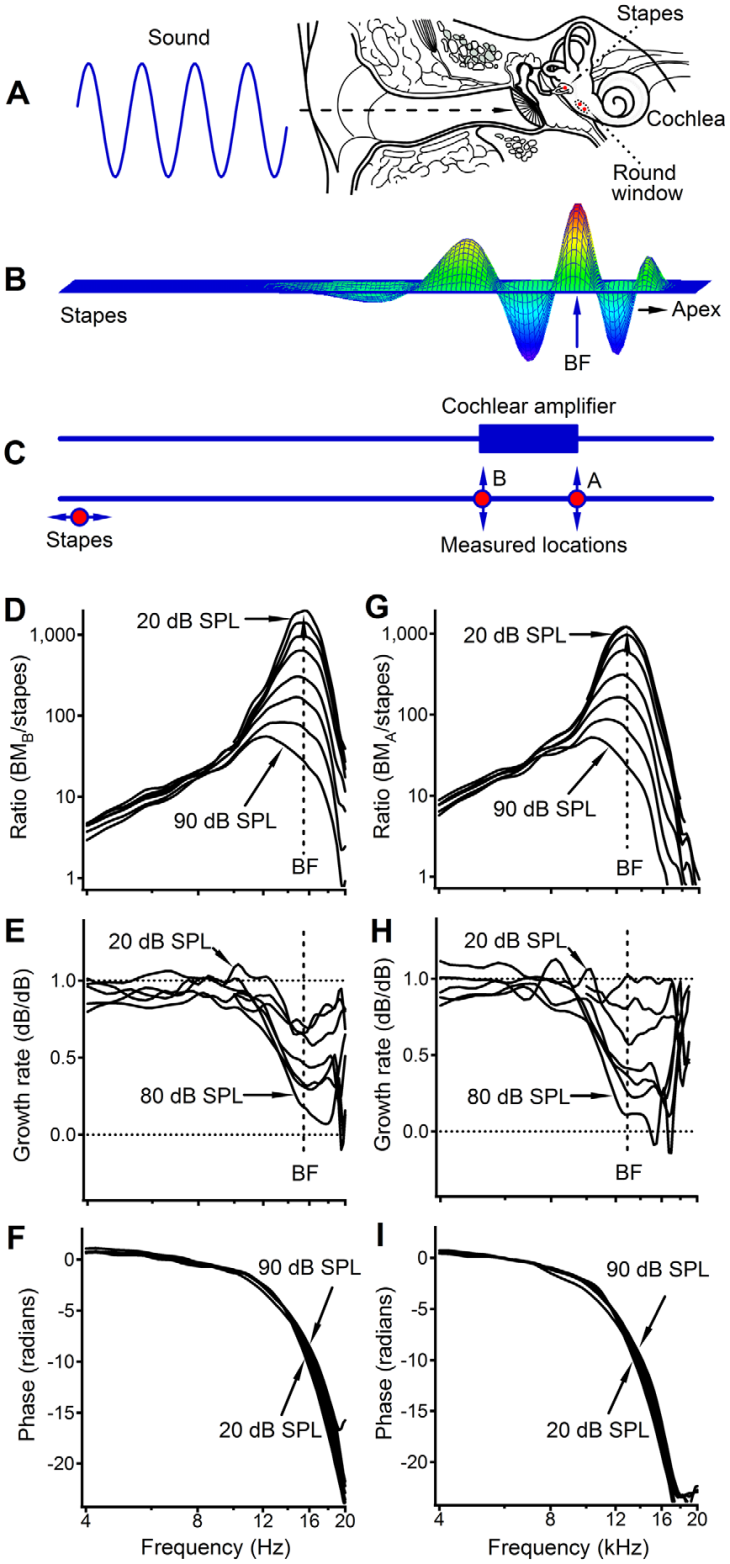
Diagrams for measuring basilar membrane vibrations. (A) Two measured locations on the BM and one on the stapes (red dots). As the wave travels from the base to its BF location (B), the cochlear amplifier increases the BM vibration at a location basal to the BF site (blue bar in panel C). The local transfer function can specifically quantify the functioning of the amplification region between positions A and B. (D) shows a sharp peak at ∼15.3 kHz at low sound levels, which was >1,000 at 20 dB SPL. As the sound level increased, the peak magnitude decreased, and the peak broadened and shifted toward ∼12.0 kHz. (E and H) Growth rates in dB/dB at the more basal (E) and apical (H) locations. (F) The phase lag progressively increased with frequency. The data in panels G–I, measured at the more apical location, are similar to those in panels D–F (allowing for a lower BF). BM_B_ and BM_A_ are BM vibration magnitudes at the measured basal and apical locations.

In the mammalian ear, incoming sounds vibrate the eardrum, propagate along the middle-ear bony chain, and enter the cochlea at the oval window (Fig. 1A). The stapes vibration at the oval window results in a pressure change in the cochlear fluid [1]. Due to impedance difference between the oval and round window this pressure wave results in a pressure difference across the cochlear partition, which initiates a forward traveling wave [2]. The cochlear traveling wave starts at the base and propagates along the basilar membrane (BM) towards the apex. As the wave travels, its amplitude gradually increases and the speed decreases. The vibration reaches the maximum at the best frequency (BF) location and then declines sharply [2]–[6] (Fig. 1B). In sensitive living cochleae, the soft sound-induced vibration at the BF location is >1,000-fold larger than that at the stapes. This ratio becomes smaller as the stimulus level increases, indicating the cochlear nonlinearity [6]–[12]. The cochlear sensitivity, sharp tuning, nonlinearity, and spontaneous otoacoustic emission have been attributed to the cochlear amplifier, a outer hair cell-based active process proposed to amplify the BM response to soft sounds [13]–[21].

The cochlear amplifier is believed to reside at an area immediately basal to the BF location [13]–[15], [21] (blue bar in Fig. 1C). As waves propagate through this region the cochlear amplifier generates energy and boosts the BM vibration consequently resulting in a peak response at an apical location (location A in Fig. 1C). The cochlear amplifier has been studied by measuring the BM transfer function [22]. The transfer function of the BM vibration is conventionally measured as the ratio of the BM to stapes vibration magnitude as a function of frequency [22]. However, the conventional transfer function is determined by the total mechanical processes from the stapes to the measured BM location, and it provides no spatial information about where the amplification occurs. In the current study, we localized the cochlear amplifier by measuring BM vibrations at two longitudinal locations (A and B in Fig. 1C) and quantifying the local transfer function of the BM between the two locations. The results show that the cochlea can increase vibration magnitude at a longitudinal region basal to the peak response location in a frequency- and level-depend manner. Thus, we conclude that the cochlea amplifier resides at a location basal to the response peak in the sensitive living cochlea.

## Results

**Figure 2:**
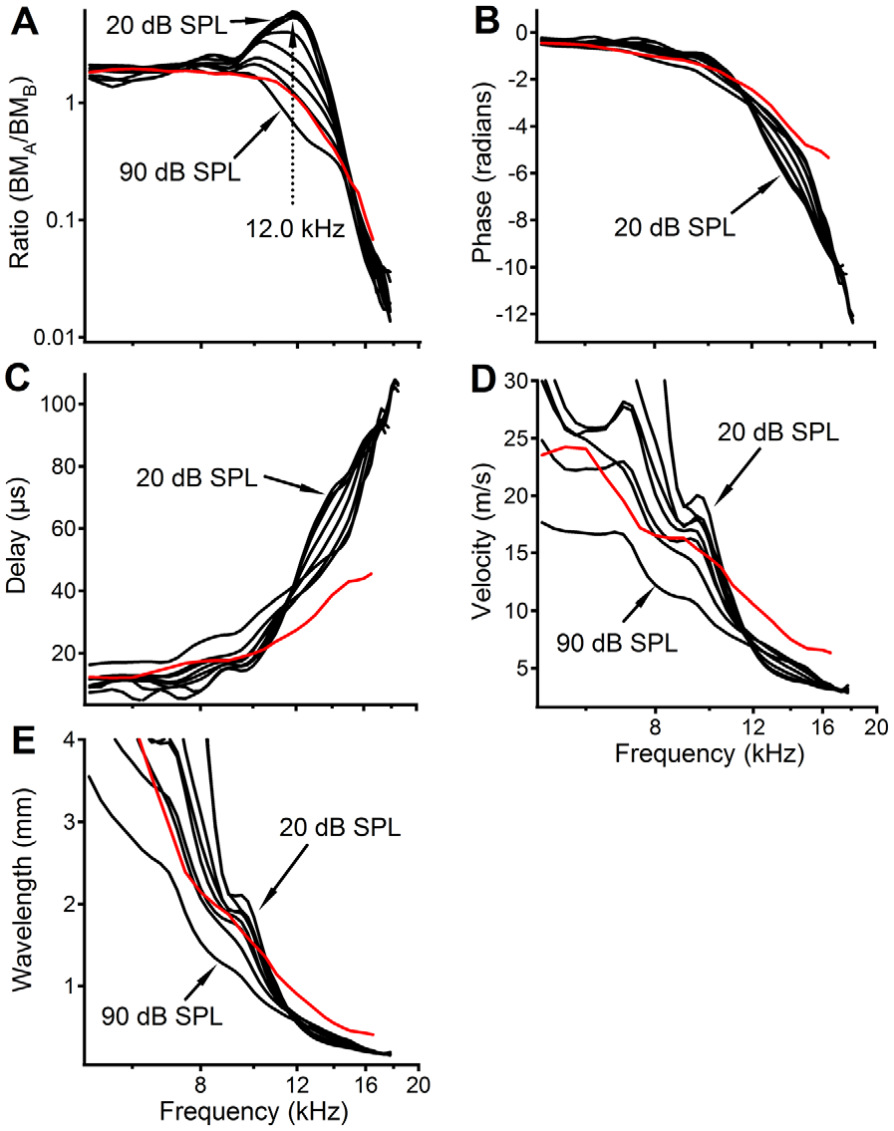
Local transfer functions, delay, velocity, and wavelength of basilar membrane vibration. (A) The response peak at ∼12.0 kHz decreased, broadened, and shifted toward low frequencies with increasing sound level. The magnitude was smallest at ∼17.0 kHz. (B) Phase response was similar to that in Figures 1F and I but with a smaller phase lag. The delay from the basal to more apical location increased with frequency (C), while the propagation velocity (D) and wavelength (E) decreased over the same frequency range. Red lines show post-mortem data measured at 40 dB SPL.

**Figure 3:**
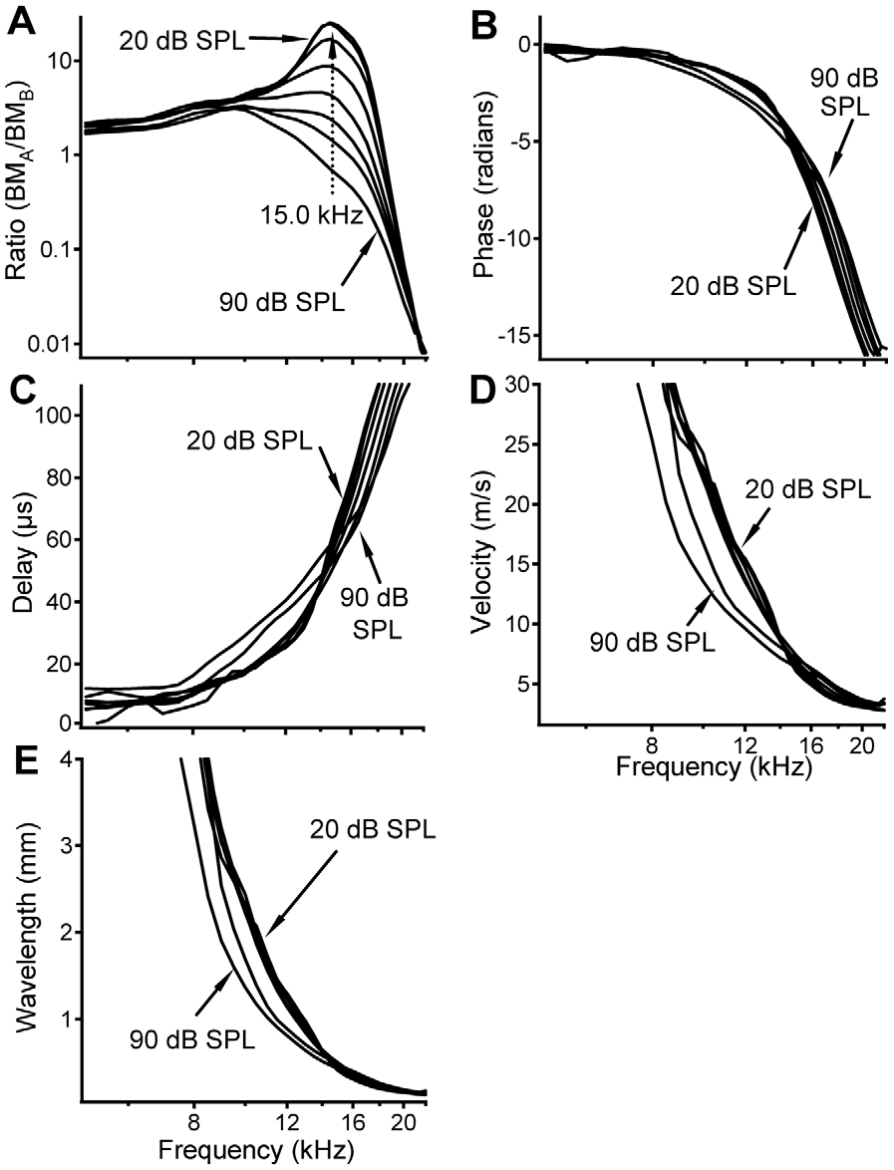
Local transfer functions, delay, velocity, and wavelength in a different sensitive cochlea. Data were collected at longitudinal locations ∼2,650 and ∼2,317 µm with ∼333 µm separation. Allowing a higher peak frequency of 15.0 kHz in panel A, the data in Figure 3 are similar to those in Figure 2, which confirm the existence of magnitude amplification and reduction over the BM region between the two measured locations.

**Figure 4:**
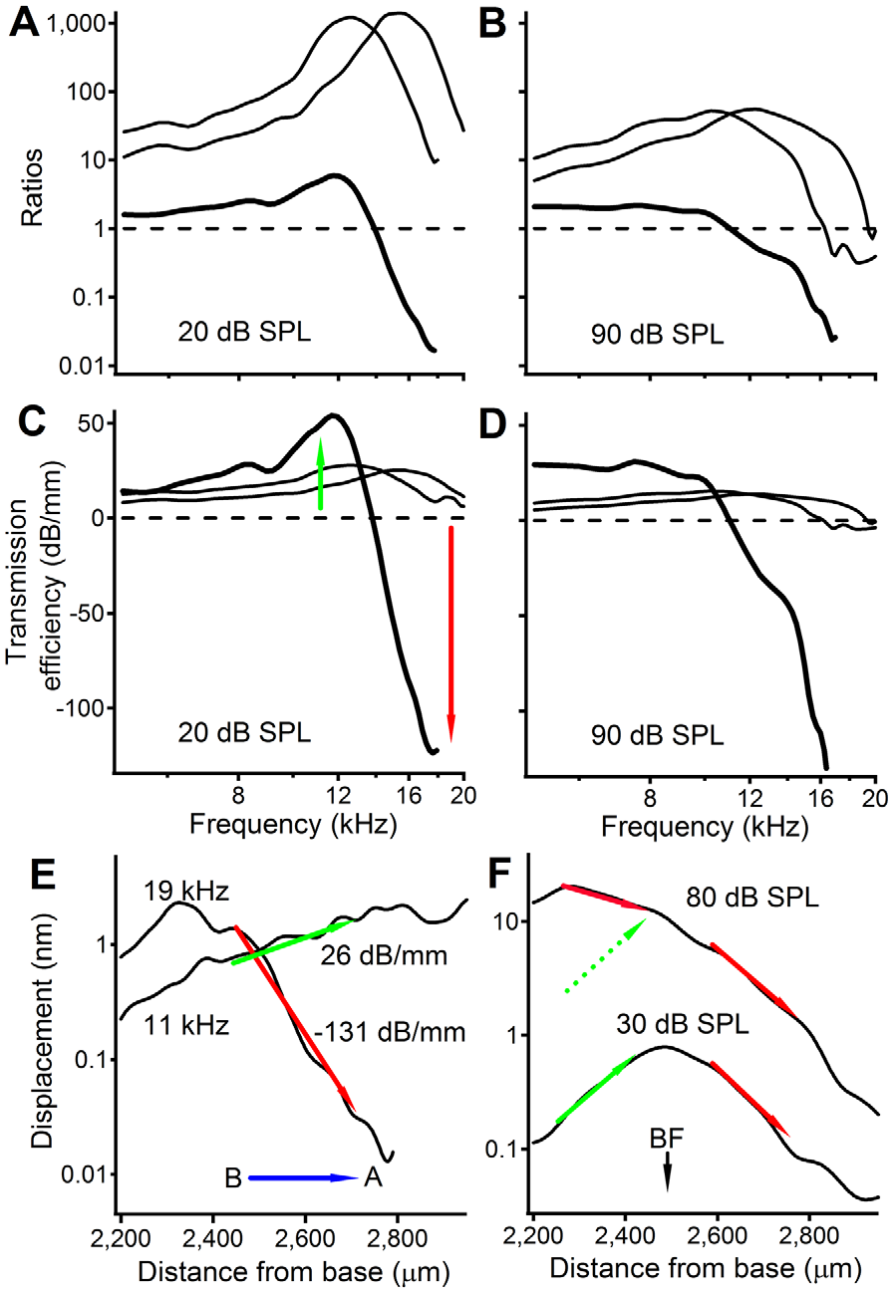
The relationship between transfer functions and the longitudinal pattern of basilar membrane vibration. In contrast to the >1,000 gain of the conventional transfer function (thin lines) at the peak frequency, the local transfer function (thick lines) shows a gain of only ∼10 at ∼12.0 kHz and ∼40 dB of reduction at ∼17 kHz in panels A. Response peaks became smaller at 90 dB SPL in panel B. (C) At 20 dB SPL, the highest transmission efficiency was >50 dB/mm at ∼12.0 kHz and the lowest efficiency was <−100 dB/mm at ∼17.0 kHz (thick line). (D) At 90 dB SPL, the response peak at ∼12.0 kHz disappeared and the minimum remained unchanged (thick line). (E) BM response to a 50 dB SPL 11.0-kHz tone increased at the rate of ∼26 dB/mm in the region between 2,450 to 2,750 µm (green arrow), while the 19.0-kHz response decreased at the rate of ∼131 dB/mm over the same distance (red arrow). (F) The increase in low-level response on the basal side of the BF location (solid green arrows) became the decrease at the high sound level (red arrow near 2,300 µm).

**Figure 5:**
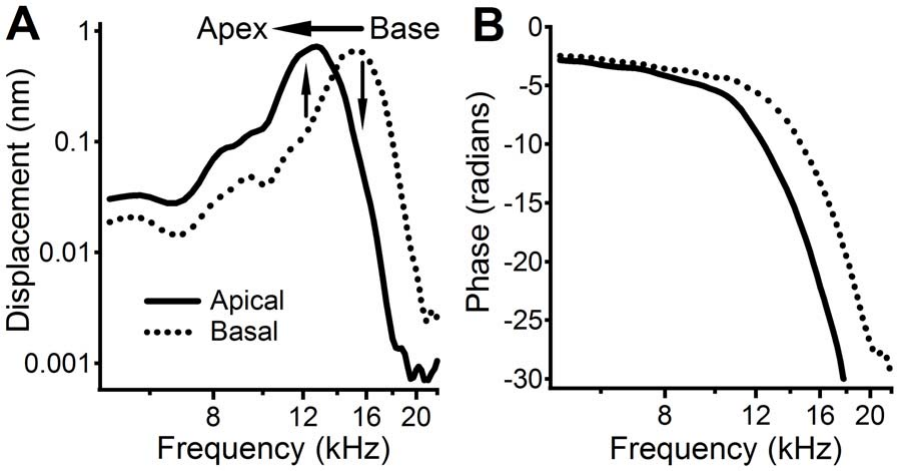
Frequency-dependent amplification and reduction and BM sharp tuning. (A) The BM response at the basal location is presented by vibration amplitude as a function of frequency (dotted curve), and that at the more apical location is shown by the solid curve. The peak frequency of the basal location was higher than that of the more apical site. As the vibration propagated from base to apex, the BM between the two measured locations increased low-frequency responses (upward arrow) and reduced high-frequency responses (downward arrow), resulting in a sharply tuned response at the apical location (solid curve). (B) Phase at the basal location (dotted curve) leaded that at the apical location (solid curve), indicating that waves propagated from base to apex at frequency-dependent speeds. Data were collected at 30 dB SPL from the same sensitive cochlea as for Figures 1 and 2.

All animals tolerated anesthesia and survived from surgery. However, because of the invasive surgery required to access the BM and the inherent vulnerability of high-frequency hearing, the data acquisition efficiency from sensitive cochleae was low. The principle constraints were high-frequency hearing loss and poor visibility of the cochlear fluid. The latter was caused by blood cells and tissue fluid in the scala tympani, which reduced the signal level and resulted in a high noise floor of the vibration measurement. Measurements with a noise floor of >0.2 µm/s were excluded from this report. The data presented in Figure 1D–I, Figure 2, Figure 4A–D, and Figure 5 were collected from one sensitive cochlea, and those in Figure 3 and Figure 4E and F were from two of five sensitive cochleae.

Conventional transfer functions and growth rates of BM vibrations in a sensitive cochlea measured at two longitudinal locations with a separation of ∼288 µm are presented in Figures 1D–I. Data in Figures 1D–F were collected at the more basal location ∼2,450 µm from the base. At low sound levels, the ratio of the BM to stapes vibration increased with frequency, peaking at ∼15.3 kHz (i.e., the BF, indicated by the vertical dashed lines in Figs. 1D and E) and then decreased rapidly at higher frequencies. At 20 and 30 dB SPL (0 dB SPL ref. 20 µPa), the BM peak response was >1,000-fold greater than the stapes vibration and decreased with increasing sound level. For an ∼3,333-fold increase in sound pressure level, from 20 to 90 dB SPL, the ratio decreased ∼100 times, which indicates a strong nonlinear compression. The compression is confirmed by the growth rates of <1 near BFs (Figs. 1E). Also, the response peak broadened and shifted to ∼12.0 kHz with increasing sound level. Figure 1D and E shows the typical features of the BM response in a sensitive living cochlea: high sensitivity, sharp tuning, and nonlinearity [6], [8]–[12], [22], [23]. The corresponding phase decreased progressively with frequency, indicating a decrease in the wave speed at high frequencies (Fig. 1F). Allowing for a lower BF, the data measured at the more apical location in Figures 1G–I were similar to those shown in Figures 1D–F.

Local transfer functions obtained from the data in Figures 1 are presented in Figures 2A and B (black curves). Figure 2A shows that below 10.0 kHz the ratios of the BM vibration amplitude at the more apical location to that at the more basal location were approximately independent of sound level and frequency. At frequencies above 10.0 kHz the ratios for low-level stimuli increased with frequency, peaking at ∼12.0 kHz and then rapidly decreasing to <1, becoming as small as ∼0.01 at high frequencies. The peak ratio at 12.0 kHz decreased with increasing sound level, becoming <1 at 90 dB SPL, while reduction near 17.0 kHz showed no significant change with the sound level. One noteworthy finding is that the peaks of the magnitude transfer functions in Figure 2A were much smaller than those of the conventional magnitude transfer function shown in Figures 1D and G. Even more striking is that the minimum ratio in Figure 2A was as small as 0.01. Since ratios >1 and <1 indicate amplification and reduction, respectively, Figure 2A indicates the cochlear partition between the two measured locations can amplify and reduce the BM vibration.

The phase transfer functions between the two measured locations in Figure 2B (black curves) show only negative values, indicating that waves arrived at the basal location earlier than at the more apical location and propagated in the apical direction [6], [24], [25]. As in Figures 1F and I, the phase lag in Figure 2B also accumulated progressively with frequency. At frequencies near 12.0 kHz, the slopes of phase curves became flatter at high sound levels, indicating an increase in wave speed.

Phase difference in Figure 2B allows quantifying the delay, velocity, and wavelength of BM vibration over the region between the two measured locations [25]. Black curves in Figure 2C show that the delay increased with frequency by as much as eight times from ∼10.0 µs at 5.0 kHz to >80 µs at 16.0 kHz. The wave propagation velocity in Figure 2D decreased with frequency, becoming as small as ∼3 m/s at ∼16.0 kHz. Similarly, the wavelength in Figure 2E decreased with frequency, becoming as small as 0.15 mm at ∼16.0 kHz. At frequencies near the peak of the local transfer function (∼12.0 kHz), the delay, velocity, and wavelength varied rapidly with frequency, indicating a strong dispersion. The variation rates decreased with increasing stimulus level. These features indicate that observed amplification was related to the degree of the dispersion of the cochlea. Post-mortem data obtained at 40 dB SPL (red lines in Fig. 2) show the absence of a response peak (Fig. 2A), less phase lag (Fig. 2B), decreased delay (Fig. 2C), and increased velocity (Fig. 2D) and wavelength (Fig. 2E) at and above 12.0 kHz.

To show the similarity of data across animals, another data set measured in a different sensitive cochlea is presented in Figure 3. Allowing a high peak frequency of 15.0 kHz in panel A, the data in Figure 3 are similar to those in Figure 2. The higher BF and longer distance between the two measured locations in Figure 3 than those in Figure 2 likely contribute to differences in the delays, velocities, and wavelengths between the two figures.

In order to show their difference, the conventional and local transfer functions measured at a low (20 dB SPL) and high (90 dB SPL) sound level are plotted together in Figures 4A and B. Although both conventional (thin lines) and local transfer function (thick line) showed response peaks, their peak frequencies were different. The peak of the local transfer function was at frequencies immediately below the BF of the more apical location. According to the cochlear frequency- location map [26], the peak response in the local transfer function indicates that the BM region between the two measured locations amplified waves maximally at the BF of the more apical location. Similarly, the magnitude reduction suggests that the same BM region attenuated waves at high frequencies above the BF of the more basal location. In contrast to conventional magnitude transfer functions (thin lines), which showed amplification only, the local transfer function in Figure 4A (thick line) indicates that magnitude amplification and reduction were present between the two measured locations. The thick solid line in Figure 4B shows the absence of a clear peak in the local transfer function at a high sound level (90 dB SPL).

For a quantitative comparison, the transmission efficiency, the magnitude change in dB per unit of the BM length, was calculated from the transfer function and the distance between the two measured BM locations or the distance from the base to a BM location (see Methods), and is presented in Figures 4C and D. At 20 dB SPL, the highest transmission efficiency (thick line in Fig. 4C) was >50 dB/mm at ∼12.0 kHz and the lowest efficiency was <−100 dB/mm at ∼17.0 kHz. At 90 dB SPL, the response peak at ∼12.0 kHz disappeared and the peak magnitude decreased from >50 dB/mm to ∼−15 dB/mm. The maximal magnitude of the transmission efficiency derived from the conventional transfer function (thin lines in Fig. 4C) was much smaller than that from the local transfer function. This magnitude difference indicates that BM vibration magnitude was amplified and reduced mainly over the distance between the two measured locations.

In order to confirm the observed magnitude amplification and reduction and to reveal their relationship with the longitudinal pattern, BM vibration was also measured as a function of longitudinal location using a scanning laser interferometer [27]. BM response to a 50 dB SPL tone at 11.0 kHz increased at the rate of ∼26 dB/mm from 2,450 to 2,750-µm place (green arrow in Fig. 4E), while the response to a 19.0-kHz tone decreased at the rate of ∼131 dB/mm over the same distance (red arrow in Fig. 4E). These frequency-dependent increases and decreases are related to the magnitude amplification and reduction as shown by the transmission efficiency derived from the local transfer function (green and red arrows) in Figure 4C. Figure 4F shows longitudinal patterns of BM responses to 16.0-kHz tones at 30 and 80 dB SPL. In contrast to the level-independent decreases on the apical side of the BF location, the increase of low-level response on the basal side (solid green arrow) became the decrease at high sound levels (red arrow near 2,300 µm). This level-dependent slope change was consistent with the absence of the response peak of the high-level transmission-efficiency curve (thick line) in Figure 4D. Thus, Figure 4 shows that observed frequency- and level-dependent amplification and reduction of BM vibration are the presentations in the frequency domain of the increase and decrease of the vibration magnitude as a function of the longitudinal location.

## Discussion

Direct measurement of the sound-induced cochlear-partition vibration has been demonstrated to be one of the most efficient approaches for studying cochlear mechanics. The cochlear traveling-wave theory was established based on direct observation of BM vibrations in the human and animal cadavers [2], [3]. Cochlear functions in the living ear, however, are vulnerable and susceptible to damage caused by measurement procedures. For maintaining normal cochlear functions, BM vibration is often measured only at a single location in living cochleae. Instead of measurements at different locations, the magnitude and phase of the vibration are measured as a function of frequency [22]. While the conventional transfer function is adequate for measuring the cumulative function from the stapes to a BM location, the local transfer function can specifically quantify the function of the BM region between the two measured locations. Because cochlear amplification is thought to be a local mechanism, the local transfer function can provide more precise and complete information on the cochlear amplifier than the conventional transfer function. Although BM vibrations were measured from more than one longitudinal location in a few previous studies [6], [11], [23]–[25], no local transfer function was reported in the literature.

The principle for measurement of the local transfer function is the same as that for measuring the conventional transfer function, with the only difference being in the input: the stapes vibration for the conventional transfer function and the BM vibration at a more basal location for the local transfer function. According to the cochlear traveling-wave theory [4], [5], BM vibration at an apical location results from vibrations at basal locations. Because the BM vibration at a single location has been used as the output for calculating the conventional transfer function [22], the BM vibration at a more basal location was used as the input for quantifying the local transfer function in this study. In fact, the vibration at an apical location is more comparable to that at a more basal location than the stapes vibration because of the structural and functional similarities at the two BM locations.

While the length of the cochlear-amplification area is not well defined, a low-level 16.0-kHz tone results in vibration over only an ∼600-µm-long region of the BM in the sensitive gerbil cochlea [27]. The longitudinal extent of the BM vibration includes a magnitude-increasing region basal to the response peak and a magnitude-decreasing region apical to the peak (the 30-dB SPL response in Fig. 4F). The length of either region is ∼300 µm, which is comparable to the distance between the two measured locations in this experiment. Thus, the local transfer function should at least partially reveal the function of the proposed cochlear amplifier that contributes to the vibration at the measured apical location. The local transfer functions in Figures 2A, and 3A, and the transmission efficiency functions in Figure 4C, show amplification of the BM vibration at frequencies immediately below the BF of the more apical location, which is confirmed by the spatial patterns of BM responses in Figures 4E and F. This frequency- and level-dependent amplification is conceptually consistent with the theory that the cochlear amplifier boosts the BM vibration at a region basal to the BF location [13]–[15] (indicated by the blue bar in Fig. 1C). However, at frequencies above the BF, the local transfer functions indicate that BM vibration magnitude is reduced, which is not evident in conventional transfer functions. This magnitude reduction is critical to sharp tuning since the input vibration at the more basal location peaks at a frequency higher than the BF of the more apical location. To achieve a sharp peak response at the apical site, low-frequency responses at the basal location need to be amplified, and high-frequency responses need to be reduced (Fig. 5A). In contrast to the current result, the amplification region for a 15-kHz tone was found to be 1.25-mm long and centered on the best-frequency place in guinea pig [11].

The observation that the response peak decreases with increasing sound level (Figs. 2A and 3A) and disappears under the postmortem condition (red line in Fig. 2A) indicates that magnitude amplification of the BM vibration likely results from the cochlear amplifier. It has been demonstrated that the hair bundle of mammalian outer hair cell can produce mechanical force as in vertebrates [19], [20], [28]–[30]. When the membrane potential of an outer hair cell changes as a result of sound-induced hair-bundle deflection, the cell body elongates or shortens due to conformational change of the membrane protein prestin [31]–[35]. By contrast, the lack of significant change with the stimulus level suggests that the magnitude reduction probably results from a passive cochlear mechanism.

## Materials and Methods

Thirty healthy young Mongolian gerbils (40–80 g) were used in this study. Animal preparation and surgical procedures were the same as described previously [25], [27], [36]. The initial anesthesia was induced by intraperitoneal injection of ketamine (30 mg/kg) followed by intramuscular injection of xylazine (5 mg/kg). The animal's head was attached to a custom-made holder with x-y-z translation and rotation capability. After a tracheotomy was performed, a ventilation tube was inserted into the trachea to maintain free natural breathing. Body temperature was maintained at 38±1°C with a servo-regulated heating blanket. Sensitivity of the cochlea was monitored by measuring the acoustically induced compound action potential through round window and neck electrodes. The animal use protocol IS00000130 was approved by the Oregon Health & Science University Institutional Animal Care and Use Committee.

The left auditory bulla was opened surgically under a surgical microscope. After the round window membrane was carefully removed, a few gold-coated glass beads (∼20 µm in diameter) were placed on the BM. Desired bead positions were achieved by adjusting the angles of the animal's head and controlling the entry point of the beads into the perilymph. The opened round window was partially covered with a thin glass cover slip to eliminate optical distortion at the surface of the cochlear fluids and to maintain low impedance of the window. The object beam of a heterodyne laser interferometer (OFV 302, Polytec, Inc., Germany) was focused on a bead on the BM through a long-working-distance objective. Reflected light with the Doppler shift from the vibrating bead was collected by the same objective and sent back to the interferometer. The voltage output of a digital decoder was proportional to the vibration velocity of the bead along the optical axis. Beads have been shown to accurately follow the BM vibration [37]. The noise floor of the measurement is <0.1 µm/s, corresponding to <0.001 nm at 15 kHz. The best frequency of the observed location was determined as the frequency with the maximum amplitude in the conventional transfer function of the BM vibration at 40 dB SPL.

A custom-written program was used to control hardware (System II, Tucker-Davis Technologies, Gainesville, FL) for signal generation and data acquisition. Tone bursts at different frequencies with 23-ms duration and 1-ms rise/fall time were generated by a D/A converter. The signals were sent to a power amplifier through a programmable attenuator and then used to drive a speaker (ER-2, Etymotic Research, Inc. Elk Grove Village, IL). A sensitive microphone (10 B+, Etymotic Research, Inc. Elk Grove Village, IL) was used to measure the sound pressure in the ear canal. The microphone-earphone probe was coupled into the external ear canal to form a closed sound field. The signals from the interferometer decoder were digitized with an A/D converter and averaged 10 to 40 times. The magnitude and phase of the vibration at the stimulus frequency were obtained by use of the Fourier transformation. The vibrations at two longitudinal locations on the BM and at the stapes were sequentially measured at sound levels from 20 to 90 dB SPL, and at frequencies from 250 Hz to 23.0 kHz in 250-Hz steps. The displacement of the BM vibration (*D*) in nanometers (nm) was obtained from recorded vibration velocity (*V*) in µm/s according to D = V/(2πf)*1,000, where *f* is frequency (Hz).

For measuring the stapes vibration, a gold-coated glass bead was placed on the anterior surface of the anterior crus of the stapes. The animal head position was adjusted to allow the laser beam access to the bead in a direction as perpendicular to the stapes footplate as possible.

The conventional transfer functions at a BM location were presented by the ratio of the BM to stapes vibration (*M_c_*) and the phase difference between the two (*φ_c_*) as a function of frequency. *M_c_* was obtained by dividing the BM vibration magnitude (*V_BM_*) by the stapes vibration magnitude (*V_st_*) (i.e., *M_c_* = *V_BM_*/*V_st_*), and *φ_c_* was calculated by subtracting the stapes phase (*φ_st_*) from the BM phase (*φ_BM_*) (i.e., *φ_c_* = *φ_BM_*−*φ_st_*).

The local transfer function magnitude (*M_L_*) was presented by the ratio of the BM vibration magnitude at the more apical location (*V_BMA_*) to that at the more basal location (*V_BMB_*) (i.e., *M_L_* = *V_BMA_*/*V_BMB_*). The corresponding phase (*φ_L_*) was obtained by subtracting the phase at the basal location (*φ_BMB_*) from that at the more apical location (*φ_BMA_*) (i.e., *φ_L_*  = *φ_BMA_*−*φ_BMB_*). Transmission efficiency of the BM (*E_T_*) was obtained at different frequencies based on *M_L_* or *M_c_* and the distance between the two BM locations or the distance from the base to a BM location (*d*) according to *E_T_* = *M_d_*/*d* or *E_T_* = *M_c_*/*d*, where *E_T_* is in dB/mm, *M_L_* and *M_c_* in dB, and *d* in mm.

The phase delay, propagation velocity, and wavelength of BM vibration over the distance between the two measured locations were derived from the local phase transfer function [25]. The phase delay from the basal to the more apical location (*t*) was calculated from the phase difference (*φ_A−B_*) and the frequency ( *f *) according to *t = −(φ_A−B_)/(2πf)*, where *t* is in s, *φ_A−B_* is in radians, and *f* is in Hz. The propagation velocity of the BM vibration (*v*) was quantified according to *v = d/t*, where *d* is the distance between two measured locations, and *t* is the delay over *d*. The distance *d* was obtained from the x, y, and z coordinates at different locations along the BM, which were measured using a positioning system consisting of a controller (ESP300) and three motorized linear translation stages (MFN25CC; Newport Corporation, Irvine CA). The wavelength of the BM wave (λ) was calculated based on the velocity (*v*) and frequency ( *f* ): *λ = v/f*, where *λ* is in m, *v* is in m/s, and *f* is in Hz.

For observing the relationship between the local transfer function and the longitudinal pattern, the BM vibration was also measured as a function of the longitudinal location in five cochleae as described previously [27], [36]. Approximately 1 mm of the BM in the first turn was exposed through the round window. The object beam of a laser interferometer was focused on the BM. The scanning paths were determined by 10 to 20 reference points using the 3-dimensional positioning system. The longitudinal scanning path was approximately underneath the second row of outer hair cells. As the longitudinal position of the laser focus spot was changed along the scanning path at the rate of 5.0 µm/s, magnitudes and phases of the BM response to a continuous tone were collected at 2 samples/s, giving a rate of 0.4 sample/µm.

## References

[1] Peterson LC, Bogert BP (1950). A dynamic theory of the cochlea.. J Acoust Soc Am.

[2] Zwislocki JJ (1953). Wave motion in the cochlea caused by bone conduction.. J Acoust Soc Am.

[3] von Békésy G (1960). Experiments in Hearing.

[4] von Békésy G (1970). Travelling waves as frequency analysers in the cochlea.. Nature.

[5] Zwislocki JJ (2002). Auditory Sound Transmission: An Autobiographical Perspective.

[6] Rhode WS (1971). Observations of the vibration of the basilar membrane in squirrel monkeys using the Mossbauer technique.. J Acoust Soc Am.

[7] Khanna SM, Leonard DG (1986). Measurement of basilar membrane vibrations and evaluation of the cochlear condition.. Hear Res.

[8] Ruggero MA, Rich NC (1991). Application of a commercially-manufactured Doppler-shift laser velocimeter to the measurement of basilar-membrane vibration.. Hear Res.

[9] Nuttall AL, Dolan DF, Avinash G (1991). Laser Doppler velocimetry of basilar membrane vibration.. Hear Res.

[10] Cooper NP, Rhode WS (1992). Basilar membrane tonotopicity in the hook region of the cat cochlea.. Hear Res.

[11] Russell IJ, Nilsen KE (1997). The location of the cochlear amplifier: spatial representation of a single tone on the guinea pig basilar membrane.. Proc Natl Acad Sci USA.

[12] Ren T, Nuttall AL (2001). Basilar membrane vibration in the basal turn of the sensitive gerbil cochlea.. Hear Res.

[13] de Boer E (1983). Power amplification in an active model of the cochlea–short-wave case.. J Acoust Soc Am.

[14] Davis H (1983). An active process in cochlear mechanics.. Hear Res.

[15] Neely ST, Kim DO (1986). A model for active elements in cochlear biomechanics.. J Acoust Soc Am.

[16] Dallos P (1992). The active cochlea.. J Neurosci.

[17] Hudspeth A (1997). Mechanical amplification of stimuli by hair cells.. Curr Opin Neurobiol.

[18] Fettiplace R (2006). Active hair bundle movements in auditory hair cells.. J Physiol.

[19] Ricci AJ, Crawford AC, Fettiplace R (2002). Mechanisms of active hair bundle motion in auditory hair cells.. J Neurosci.

[20] Hudspeth AJ (2008). Making an effort to listen: mechanical amplification in the ear.. Neuron.

[21] Ren T, He W, Gillespie PG (2011). Measurement of cochlear power gain in the sensitive gerbil ear.. Nat Commun.

[22] Robles L, Ruggero MA (2001). Mechanics of the mammalian cochlea.. Physiol Rev.

[23] He W, Nuttall AL, Ren T (2007). Two-tone distortion at different longitudinal locations on the basilar membrane.. Hear Res.

[24] Rhode WS, Recio A (2000). Study of mechanical motions in the basal region of the chinchilla cochlea.. J Acoust Soc Am.

[25] He W, Fridberger A, Porsov E, Grosh K, Ren T (2008). Reverse wave propagation in the cochlea.. Proc Natl Acad Sci USA.

[26] Greenwood DD (1990). A cochlear frequency-position function for several species–29 years later.. J Acoust Soc Am.

[27] Ren T (2002). Longitudinal pattern of basilar membrane vibration in the sensitive cochlea.. Proc Natl Acad Sci USA.

[28] Martin P, Hudspeth AJ (1999). Active hair-bundle movements can amplify a hair cell's response to oscillatory mechanical stimuli.. Proc Natl Acad Sci USA.

[29] Hudspeth AJ, Choe Y, Mehta AD, Martin P (2000). Putting ion channels to work: mechanoelectrical transduction, adaptation, and amplification by hair cells.. Proc Natl Acad Sci USA.

[30] Kennedy HJ, Evans MG, Crawford AC, Fettiplace R (2003). Fast adaptation of mechanoelectrical transducer channels in mammalian cochlear hair cells.. Nat Neurosci.

[31] Brownell WE, Bader CR, Bertrand D, de Ribaupierre Y (1985). Evoked mechanical responses of isolated cochlear outer hair cells.. Science.

[32] Dallos P, Zheng J, Cheatham MA (2006). Prestin and the cochlear amplifier.. J Physiol.

[33] Zheng J, Madison LD, Oliver D, Fakler B, Dallos P (2002). Prestin, the motor protein of outer hair cells.. Audiol Neurootol.

[34] He DZ, Jia S, Dallos P (2003). Prestin and the dynamic stiffness of cochlear outer hair cells.. J Neurosci.

[35] Ashmore J (2008). Cochlear outer hair cell motility.. Physiol Rev.

[36] Ren T (2004). Reverse propagation of sound in the gerbil cochlea.. Nat Neurosci.

[37] Cooper NP (1999). Vibration of beads placed on the basilar membrane in the basal turn of the cochlea.. J Acoust Soc Am.

